# Nanocarriers for intracellular co-delivery of proteins and small-molecule drugs for cancer therapy

**DOI:** 10.3389/fbioe.2022.994655

**Published:** 2022-09-06

**Authors:** Zhihong Cheng, Yongshuang Li, Duoyi Zhao, Wei Zhao, Meng Wu, Weilin Zhang, Yan Cui, Peng Zhang, Zhiyu Zhang

**Affiliations:** ^1^ Department of Orthopedics, The Fourth Affiliated Hospital of China Medical University, Shenyang, China; ^2^ Key Laboratory of Polymer Ecomaterials, Changchun Institute of Applied Chemistry, Chinese Academy of Sciences, Changchun, China; ^3^ Department of General Surgery, The Fourth Affiliated Hospital of China Medical University, Shenyang, China

**Keywords:** nanocarriers, proteins, small-molecule drugs, intracellular co-delivery, cancer therapy

## Abstract

In the past few decades, the combination of proteins and small-molecule drugs has made tremendous progress in cancer treatment, but it is still not satisfactory. Because there are great differences in molecular weight, water solubility, stability, pharmacokinetics, biodistribution, and the ways of release and action between macromolecular proteins and small-molecule drugs. To improve the efficacy and safety of tumor treatment, people are committed to developing protein and drug co-delivery systems. Currently, intracellular co-delivery systems have been developed that integrate proteins and small-molecule drugs into one nanocarrier *via* various loading strategies. These systems significantly improve the blood stability, half-life, and biodistribution of proteins and small-molecule drugs, thus increasing their concentration in tumors. Furthermore, proteins and small-molecule drugs within these systems can be specifically targeted to tumor cells, and are released to perform functions after entering tumor cells simultaneously, resulting in improved effectiveness and safety of tumor treatment. This review summarizes the latest progress in protein and small-molecule drug intracellular co-delivery systems, with emphasis on the composition of nanocarriers, as well as on the loading methods of proteins and small-molecule drugs that play a role in cells into the systems, which have not been summarized by others so far.

## 1 Introduction

Cancer is the leading cause of death worldwide, and the treatment of cancer remains one of the most challenging problems (DeSantis et al., 2014; Ward et al., 2014; Zheng et al., 2021a). Over the past few decades, the clinical treatment of cancer mainly depends on the surgery, radiotherapy, and chemotherapy (Hu J et al., 2016). In recent years, the new tumor treatment strategies have also made great progress in the clinic (Sanchez-Garcia et al., 2016; Chudy et al., 2018; Li et al., 2022). Protein-based therapies play an important role in biomedicine because of their pharmacological action and highly selective biological activity (Mitragotri et al., 2014; Su et al., 2018; Liu et al., 2019). However, proteins have unfavorable physical and chemical properties, such as fragile tertiary structure, large molecular size, and poor membrane permeability, which make the efficiency of intracellular protein delivery very low (Qin et al., 2019). And so far, protein delivery system is mainly focused on extracellular targets, but there are many potential applications that require protein delivery into the cytoplasm (Leader et al., 2008; Cheng et al., 2018; Scaletti et al., 2018; Shao et al., 2018). Protein-based therapies for clinical application are currently limited to those that function outside cells, such as cytokines and monoclonal antibodies. Protein biologics with intracellular anticancer activity have not been widely studied for clinical application due to poor cell membrane permeability.

Due to the complexity of cancer mechanism, the use of a single anticancer agent for tumor treatment may be insufficient, so the combined use of a variety of therapeutic agents to enhance the anti-tumor effect has been investigated (Hu Q et al., 2016; Chen et al., 2017; Yang and Ding 2022). The combined therapy with a variety of free anticancer agents has stronger anticancer effect than a single anticancer agent, but it also has some limitations, such as short blood circulation time, poor tumor selectivity, insufficient tumor accumulation, severe side effects and so on (Thao et al., 2015; You et al., 2018). With the development of nanocarriers, a variety of anticancer agents are loaded into a single carrier to improve and unify the pharmacokinetics, enhance tumor selectivity and drug accumulation at the tumor sites, minimize side effects and optimize the therapeutic effects (Lee et al., 2011; Wu et al., 2015; Lin et al., 2017; Wei L et al., 2021; Yang et al., 2021). Multiple co-delivery systems loaded with antineoplastic drug combinations have been developed to treat tumors, such as two chemotherapeutics, chemotherapeutic and gene, chemotherapeutic and protein, etc. (Yin et al., 2018; Zhou et al., 2018; Gao et al., 2019; Wan et al., 2021) Among them, it is relatively difficult to load small-molecule drugs and macromolecular proteins together in the nanocarriers (Fumoto and Nishida 2020). Because small-molecule drugs and proteins have unique physical and chemical properties, such as size, hydrophilic/hydrophobic properties, charge properties, specificity, stability and cell membrane permeability (Peng et al., 2019). The development of monotherapy based on multiple drug delivery techniques of small-molecule drugs or proteins is undoubtedly a very active research field of cancer therapy, but several or more drugs in combination therapy can cause the death of tumor cells by targeting different apoptotic pathways at the same time, resulting in a synergistic anticancer effect. It can also reduce the dosage of drugs and reduce side effects, which shows greater potential as an alternative to monotherapy (Lehár et al., 2009; Hu et al., 2010; Hu and Zhang 2012).

Many biological drugs work by binding to intracellular targets, so intracellular delivery is required (He et al., 2020). Therefore, to achieve the intracellular co-delivery of proteins and small-molecule drugs, we should first consider the inherent properties of proteins and small-molecule drugs, which have distinct physical and chemical properties, hindering their integration into a single nanocarrier (Kim et al., 2015). Unlike extracellular proteins, the intracellular proteins are usually encapsulated in the endosome, so they are degraded in lysosomes, resulting in very low therapeutic efficacy (Kaczmarczyk et al., 2011; Fu et al., 2014). Most traditional nanocarriers are internalized by endocytosis, and then through endocytosis escape to achieve intracellular delivery of biopharmaceuticals (He, Xing, Wang, Wu, Wu, Guo and Mitragotri 2020). Moreover, another challenge is the system stability of the co-delivery system, as the early release of cargoes in the blood may lead to adverse reactions and reduced tumor accumulation through enhanced permeability and retention of (EPR) effects (Mura et al., 2013). Nanoparticles need to stay in systemic circulation for a long time and are stable for plasma dilution, regulation, and clearance of the reticuloendothelial system (Liu et al., 2014; Zhang R et al., 2018). These challenges must be taken into account in the rational design of a co-delivery system.

**FIGURE 1:**
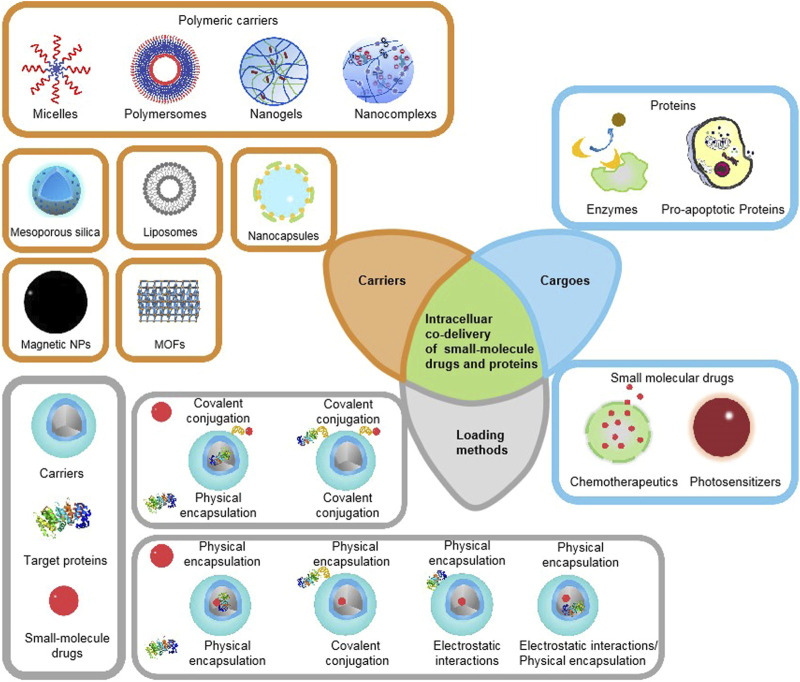
Schematic illustration of existing therapeutic proteins, small-molecule drugs, major categories of co-delivery systems, and commonly used loading/encapsulation strategies, as discussed in this Review.

This review introduces the latest development of various delivery carrier systems for intracellular co-delivery of small-molecule drugs and proteins, such as nanocapsules, liposome-based nanocarriers, polymeric nanocarriers, silica-based nanocarriers, metal−organic frameworks, and other nanocarriers, and focuses on how various proteins and small molecules are concurrently loaded into the delivery systems, as summarized in Figure 1. A recent overview of strategies for intracellular co-delivery of proteins and small-molecule drugs can provide clues about how to load multiple agents with different physicochemical properties into one single nanocarrier, and help to develop new intracellular co-delivery systems to achieve improved tumor treatment effects and reduced side effects.

## 2 Nanocarriers for intracellular co-delivery of proteins and small-molecule drugs

Due to the different biological distribution behaviors of macromolecules and small molecules, nanocarriers are the preferred drug delivery method to realize the synchronous biological distribution of proteins and drugs *in vivo* (Chiper et al., 2018; Peng et al., 2019). Therefore, in recent years, there have been some delivery systems for co-delivery of small-molecule drugs and proteins to tumor cells, by applying different ligands to locate the required subcellular compartments, such as cytoplasm, nuclei, and mitochondria. This enables targeted intracellular co-delivery of small-molecule drugs and proteins, allowing the simultaneous release of two or more drugs in a controlled manner (Biju 2014; Zhong et al., 2015; Wei Q et al., 2021). Representative carrier types and combination strategies are presented in Table 1. The covalent bonds applied for the loading of proteins or small-molecule drugs into intracellular co-delivery systems are summarized in Table 2.

**TABLE 1:** Summary of recently reported intracellular co-delivery nano-systems of proteins and drugs for tumor treatment.

Large categories of nanocarriers	Detailed categories of nanocarriers	Therapeutic proteins/Loading mode	Small-molecule drugs/Loading mode	Ref.
Nanocapsules	Nanocapsules	CASP3/Electrostatic interactions	PTX/Physical encapsulation	Kim et al. (2015)
	Chitosan nanocapsules	CASP3/Electrostatic interactions	PTX/Physical encapsulation	Wu et al. (2017)
	Dual-cargo nanocapsules	RNase A/Electrostatic interactions	Dox/Covalent conjugation	Liew et al. (2021)
Liposome-based nanocarriers	Lipid-calcium carbonate nanoparticles	Superoxide dismutase/Electrostatic interactions or hydrophobic interaction	PTX/Physical encapsulation	Peng et al. (2019)
	Liposomes	CAT/Physical encapsulation	Cisplatin/Covalent conjugation	Zhang et al. (2017)
	Dual loaded liposomal carriers	Ran-RCC1 inhibitory peptide/Physical encapsulation	Dox/Physical encapsulation	Haggag et al. (2020)
Polymeric nanocarriers	Triblock copolymer	RNase A/Covalent conjugation	Dox/Hydrophobic interaction	Zhang et al. (2020)
	Copolymer	CRISPR-Cas9 ribonucleoprotein/Covalent conjugation	Ce6/Hydrophobic interaction	Deng et al. (2020a)
	Polymeric Nanogels	β-galactosidase/Electrostatic interactions	DiI/Hydrophobic interaction	González-Toro et al. (2012)
	Polymersomes	Exogenous proteins/Electrostatic interactions and/or hydrophobic interaction	Dox/Hydrophobic interaction	Liu et al. (2010)
	Polymeric Nanocomplex	RNase A/Covalent conjugation	Oxaliplatin/Covalent conjugation	Lim et al. (2019)
	Multi-functional nanocarrier	RNase A/Covalent conjugation	Hematoporphyrin/Covalent conjugation	He et al. (2018)
Silica-based nanocarriers	Mesoporous silica nanoparticles	Cyt c/Covalent conjugation	Dox/Physical encapsulation	Pei et al. (2018)
	Silica nanoparticles	HAase/Physical encapsulation	Dox/Covalent conjugation	Chen et al. (2018)
	Large-pore mesoporous silica	RNase A/Physical encapsulation	DSP/Covalent conjugation	Teng et al. (2021)
	Multistage responsive nanoparticles	CAT/Physical encapsulation	Ce6/Covalent conjugation	Yang et al. (2018)
	Mesoporous silica nanoparticles	CRISPR-Cas9/Covalent conjugation	Axitinib/Physical encapsulation	Liu et al. (2020)
Metal−Organic Framework	Co-delivery nanoplatforms	Cyt c/Physical encapsulation	Ce6/Physical encapsulation	Ding et al. (2020)
Other nanocarriers	Magnetic nanoparticles	Trypsin/Covalent conjugation	QCy7/Covalent conjugation	Xu et al. (2019)
	Nano-Self-Assembly	TCS/Electrostatic interactions	ABZ/Physical encapsulation	Tang et al. (2017)
	Protein–polymer conjugates	Bovine serum albumin/Covalent conjugation	immune-modulators/Hydrophobic interaction	Vanparijs et al. (2015)
	Upconversion nanocrystal-dendrimer composite	CAT/Electrostatic interactions	Ce6/Hydrophobic interaction	Liang et al. (2020)
	catalase-encapsulated hyaluronic-acid-based nanoparticles	CAT/Covalent conjugation	Ce6/Supramolecular encapsulation	Phua et al. (2019)
	DNA nanoassemblies	CAT/Physical encapsulation	Porphyrin photosensitizer/Intercalation	Pan et al. (2020)

**TABLE 2:** Summary of covalent bonds applied for the loading of proteins or small-molecule drugs into intracellular co-delivery systems.

	Cargoes	Types of covalent conjugations	Release modes	Ref.
Proteins	RNase A	RNBC and then through phenylboronic acid–catechol interactions	ROS-triggered release	Zhang et al. (2020)
	CRISPR-Cas9 RNP	His-tagged Cas9 RNP	Near infrared-triggered release and reductant-triggered release	Deng et al. (2020b)
		Disulfide bonds	GSH-triggered release	Liu et al. (2020)
	Cyt c	Boronic ester bonds	ROS-triggered release	Pei et al. (2018)
	Trypsin	Trypsin conjugated with phenylboronic acid	H_2_O_2_-triggered release	Xu et al. (2019)
	CAT	β-cyclodextrin was first functionalized onto HA, followed by conjugation with CAT	—	Phua et al. (2019)
Small-molecule drugs	Cisplatin (IV) pro-drug	Pt (IV)-liposomes	Redox-triggered release	Zhang et al. (2017)
	Oxaliplatin (IV) pro-drug	PEI-oxliPt (IV)	Redox-triggered release	Lim et al. (2019)
	Hematoporphyrin	HA-HP	—	He et al. (2018)
	Dox	HA-DOX	Hyaluronidase-triggered release	Chen et al. (2018)
		BB-DOX	GSH/ROS-triggered release	Liew et al. (2021)
	Ce6	Covalently conjugated to APTES	—	Teng et al. (2021)
	Cisplatin pro-drug	Covalently conjugated to APTES	—	Yang et al. (2018)
	QCy7	QCy7 conjugated with phenylboronic acid	H_2_O_2_-triggered release	Xu et al. (2019)

### 2.1 Nanocapsules

In recent years, polymeric nanocapsules have aroused great interest in the field of drug sustained-release due to the advantages of core-shell structure. Compared with polymeric nanospheres, the solid/oil core of nanocapsules can effectively improve the drug loading efficiency and reduce the polymeric matrix content of nanoparticles (Raffin Pohlmann et al., 2002; Wang et al., 2021). The encapsulated payload can be isolated from the tissue environment through the polymeric shell, avoiding degradation or sudden release caused by pH, temperature, enzymes, and other factors (Trindade et al., 2018). By loading the drug into the polymeric nanocapsules, the drug can be protected from the destruction or degradation of the biological environment. At the same time, it can also reduce the side effects of drugs on healthy tissues (Deng et al., 2020a). The core of polymeric nanocapsules can be made from not only a hydrophobic core but also from a hydrophilic core to deliver hydrophilic molecules, such as hydrophilic anticancer drugs gemcitabine hydrochloride (Cosco et al., 2015) and doxorubicin (Vrignaud et al., 2013), water-soluble protein albumin (Li et al., 2005).

**FIGURE 2:**
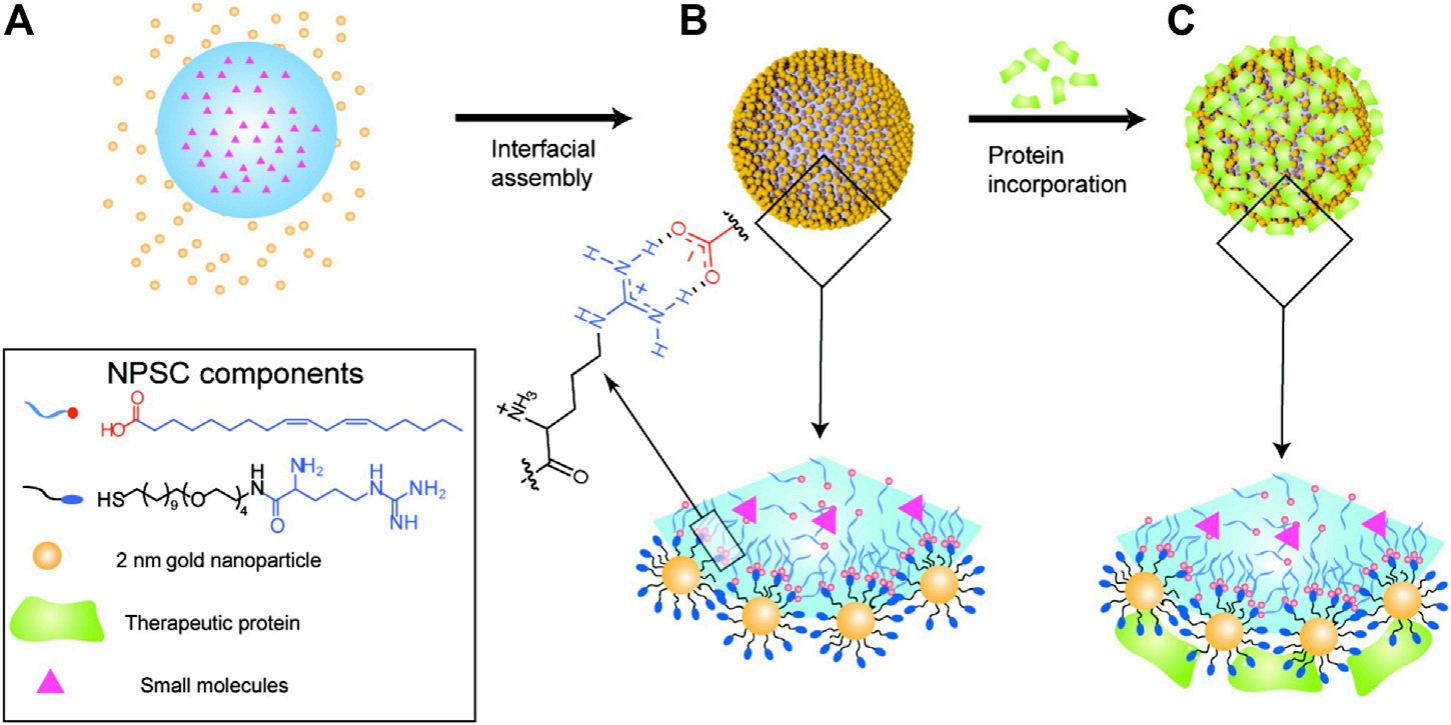
NPSC fabrication. **(A)** Template droplets (with or without PTX) were generated using an amalgamator. **(B)** Droplets were transferred to Arg-AuNP solution to generate NPSCs. **(C)** Incorporation of CASP3 or transferrin (control) onto the NPSC surface. Reproduced with permission from Ref. (Kim et al., 2015).

However, in order to realize the co-delivery of proteins and drugs, nanocapsules need to be further modified. For example, M. Rotello and his colleagues developed nanoparticle-stabilized capsules (NPSCs) that could be used to deliver proteins directly into the cytoplasm, and later they used the nanocapsules to deliver small-molecule drugs and proteins together (Tang et al., 2013; Kim et al., 2015). The paclitaxel (PTX) was first dissolved in linoleic acid to prepare the nanocapsules, which were then made into emulsions using a commercial amalgamator. Arginine ligand functionalized gold nanoparticles (Arg-AuNPs) were assembled on the surface by electrostatic attraction. The negatively charged apoptosis-inducing protein caspase-3 (CASP3) was loaded into the NPSC shells through the electrostatic interaction with Arg-AuNPs, and the mitotic spindle assembly inhibitor, PTX, was loaded into the nanocapsules through physical encapsulation (Figure 2). Finally, CASP3 and PTX were delivered into the cells and played a synergistic effect. Apoptosis study and *in vitro* cell viability measurement showed that NPSC co-delivery of PTX and CASP3 compared with NPSC loaded with PTX or CASP3 alone led to a higher percentage of HeLa cell apoptosis, indicating a higher killing rate. The final results of the Chou-Talalay analysis showed that PTX and CASP3 had an overall synergistic effect on tumor cells (Kim et al., 2015). Although the nanoparticles stabilized capsules have the advantages of negative surface charge, moderate particle size, and improved protein stability, there are still some shortcomings, such as an additional non-fouling shell may be required for prolonged blood circulation, and the metabolism of gold nanoparticles *in vivo* requires further study (He et al., 2016).

In addition, in the field of nanoscience, chitosan nano-polymers have become potential carriers because of their biodegradability and biocompatibility. The easy modification of chitosan nano-polymers and the versatility of drug delivery have attracted researchers to combine chemotherapeutics, proteins, and genetic drugs to target tumor cell-specific therapeutics (Shanmuganathan et al., 2019). Chitosan forms polycations by protonation of amino groups under the condition of low pH, which has high affinity and strong targeting to negatively charged cell membranes (Dutta et al., 2003; Fu et al., 2016). The hydrophobic drug paclitaxel can be loaded into LA droplets, and the positively charged recombinant human caspase-3 stabilizes the optimized Arg-CS nanocapsules (Arg-CNCS) on its negative surface (Wu et al., 2017). *In vitro* anti-tumor experiments showed that the co-administration system for co-delivery of paclitaxel and recombinant human caspase-3 showed lower IC_50_ values and higher apoptosis rates compared with the control group. Macromolecular proteins can be loaded by electrostatic interaction with polymeric nanoparticles with opposite charges, and small-molecule drugs can be combined through covalent modification to realize the co-delivery of proteins and small-molecule drugs.

**FIGURE 3:**
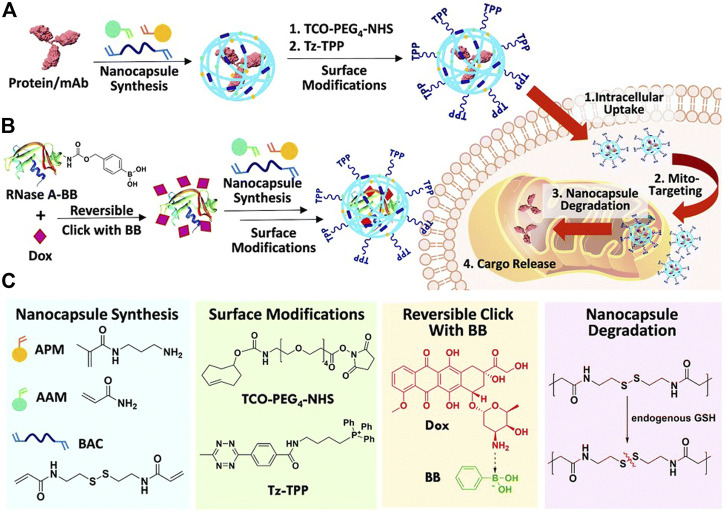
**(A)** Scheme summarizing the preparation of TPP–modified protein nanocapsules, intracellular uptake, TPP-directed mitochondrial accumulation, mitochondria GSH-triggered cargo release, and finally target binding/engagement. **(B)** Scheme showing co-encapsulation of phenyl boronic acid (BB)-modified RNase A/Doxorubicin (Dox) in a dual-cargo nanocapsule. **(C)** Chemical structures of reagents used. Reproduced with permission from Ref. (Liew et al., 2021).

The application of dual-cargo nanocapsules in mitochondrial targeting therapy with a protein and small-molecular drug (ribonuclease A/doxorubicin) was reported for the first time (Liew et al., 2021). Ribonuclease A (RNase A) was first modified with phenyl boronic acid (BB) and then clicked with doxorubicin (Dox) to form RNase A-BB-Dox conjugate, which was then crosslinked with monomers acrylamide (AAM), N-(3-aminopropyl) methacrylamide (APM) and N, N′-bis(acryloyl)cystamine (BAC) to form protein nanocapsules. Then the surface was modified with *trans*-cyclooctene (TCO)-PEG_4_-NHS due to the positive charge introduced by the APM monomer, and finally functionalized with tetrazine triphenylphosphosphonium (Tz-TPP) *via* a Tz-TCO click (Figure 3). TPP is a kind of lipophilic cation, which can target the highly negative charge site of the mitochondrial membrane, thus possessing mitochondrial targeting capability.

### 2.2 Liposomal nanocarriers

The liposome is a spherical structure formed by one or more concentric lipid bilayers around the discrete water space. Compared with other drug delivery systems, liposomes exhibit several different characteristics, including biocompatibility, non-immunogenicity, self-assembly ability, loading hydrophilic and hydrophobic reagents and improving their solubility, carrying large drug payloads, and protecting encapsulation agents from external media, reducing the toxicity of encapsulation agents and the ability to expose sensitive tissues to toxic drugs and to have site-specific targeting and improve permeability to tissues (Zununi Vahed et al., 2017). Due to their special structure, liposomes can transfer several bioactive compounds and macromolecules (such as DNA, peptides, proteins, and imaging agents) in lipid bilayers (hydrophobic molecules) and lumens (hydrophilic molecules) (Naseri et al., 2015). When different drugs are co-loaded into a liposome formulation, the pharmacokinetics and biological distribution of drugs can be improved by spatiotemporal co-administration, so that they have a specific activity in the tumor site (Mo et al., 2014).

The charge and hydrophobic interaction between lipids and proteins can load proteins into lipid nanoparticles (Wang et al., 2014). Koyo Nishida and his colleagues hypothesized that a certain proportion of the mixture of cationic lipids and neutral lipids would interact with lipophilic drugs and proteins at the same time, followed by the formation of nanoparticle skeletons (Peng et al., 2019). A complex nanoparticle consisting of lipids, calcium carbonate, and RGD peptide ligands was made by Nishida and his team to achieve intracellular co-delivery of superoxide dismutase (SOD) and PTX. They used a “one-step” ethanol injection method to prepare polyethylene glycol nanoparticles encapsulating proteins and drugs and found that when the weight ratio of EPC (Egg phosphatidylcholine)/DOTAP (1,2-dioleoyl-3-trimethylammonium-propane) was 10:1, the performance and entrapment efficiency of the nanoparticles were the best. Finally, the spherical nanoparticles with a diameter of about 130 nm were obtained by scanning electron microscope and dynamic light scattering. The formation of nanoparticles is mainly based on the electrostatic and hydrophobic interaction between proteins and lipids. The cytotoxicity of free conjugates and nanoparticles in colon26 tumor cells showed that the combination of SOD and PTX had stronger cytotoxicity than SOD and PTX alone. The results of the biodistribution study in mice with transplanted colon cancer showed that the drug delivery *via* nanoparticles was superior to free drugs in inhibiting tumor growth. The addition of SOD dismutase further increased the level of hydrogen peroxide in PTX-treated tumor cells and enhanced the cytotoxicity *in vitro*. The synchronous biological distribution of the SOD/PTX combination and the targeting of RGD modified nanoparticles to tumor areas are considered as the reasons for significant anti-tumor effects and low toxicity.

In a recent work by Liu and their team, it was found that cisplatin (IV) pre-drug coupled phospholipids can easily form Pt (IV) liposomes together with other commercial lipids (Feng et al., 2016). In order to enhance radiotherapy and chemotherapy for tumors, the antioxidant enzyme catalase (CAT) was encapsulated in cisplatin (IV)-prodrug coupled phospholipid liposomes to form CAT@Pt (IV)-liposomes (Zhang et al., 2017). Water-soluble CAT was encapsulated inside the liposomes formed with cisplatin (IV) pro-drug conjugated 1,2-distearoyl-sn-glycero-3-phosphoethanolamine (Pt (IV)-DSPE),1,2-dipalmitoyl-sn-glycero-3-phosphocholine (DPPC), cholesterol, and polyethylene glycol (PEG) conjugated DSPE. CAT in CAT@Pt (IV)-liposome has preserved and well-protected enzyme activity. After intravenous injection, the CAT@Pt (IV)-liposome can effectively accumulate in the tumor. The experiment of combined radiotherapy and chemotherapy showed that the combination of CAT@Pt (IV) and liposome had the best inhibitory effect on tumor growth.

It is feasible to co-transport peptides and drugs into cells through lipid carriers. Using the thin-film rehydration technique, a liposome nanocarrier was prepared to transfer Ran-RCC1 inhibitory peptide (Ran-IP) and Dox into breast cancer cells. By changing the pH value of the dispersion medium, combined with a number of peptides and the loading amount of Dox, the best double-loaded liposomes were selected in the preparation of polypeptide liposomes. The optimal loading rate of peptides was 85%, the particle size was about 80 nm, and the release of peptides and Dox could be maintained for 3 days. The results of *in vivo* experiments confirmed that the anticancer activity of double-loaded liposomes was enhanced, and the tumor growth inhibition rate was significantly increased after treatment with a gradient dose of double-loaded liposomes. *In vitro* cell activity studies showed that compared with free peptides and free Dox, peptides liposomes had a better cytotoxic effect. The final toxicity test showed that the combined drug delivery system was safer for liver and kidney tissue than free Dox (Haggag et al., 2020).

### 2.3 Polymeric nanocarriers

Among many nanomaterials, polymeric nanoparticles have the characteristics of adjustable chemical structure, simple synthesis, controllable relative molecular weight, low cost, environmental friendliness, easy coupling of functional groups (after functionalization), biocompatibility and biodegradability, it has important application value in the field of drug sustained release (Li et al., 2018; Jiang et al., 2022). The hydrophobic interaction between drug and polymer is the main driving force of drug entrapment. However, the hydrophobic interaction is non-specific and occurs between free drug molecules as well. Therefore, unwanted drug aggregation will occur in the process of self-assembly, which reduces the drug loading efficiency and heterogeneity of the preparation (Lv et al., 2018). It is reported that polymers, especially cationic polymers, have high cell uptake and intimal escape ability (Yang et al., 2015; Hu J et al., 2016). Therefore, the strategy of intracellular co-delivery of proteins and drugs based on polymer design is very promising.

#### 2.3.1 Polymeric micelles

Polymeric micelles are generally assembled by amphiphilic copolymers, in order to generate a nano-sized core/shell (Wang et al., 2011). The hydrophobic core of the micelles promotes the dissolution and encapsulation of small**-**molecule hydrophobic drugs. Hydrophilic molecules are usually carried by micelles through physical interaction or covalent conjugation (He et al., 2016). Hydrophilic Dox and hydrophobic paclitaxel (TAX) are co-delivered with amphiphilic methoxy PEG-PLGA copolymeric nanoparticles (Wang et al., 2011). However, these two drugs are both small-molecule drugs. If the hydrophobic small-molecule drugs are replaced with hydrophobic macromolecular proteins, there may be more challenges to be faced. Polymeric micelles are widely used in the delivery of hydrophobic drugs, but their drug loading, colloid uniformity, preparation stability, and drug release performance are not ideal (Lv et al., 2018).

**FIGURE 4:**
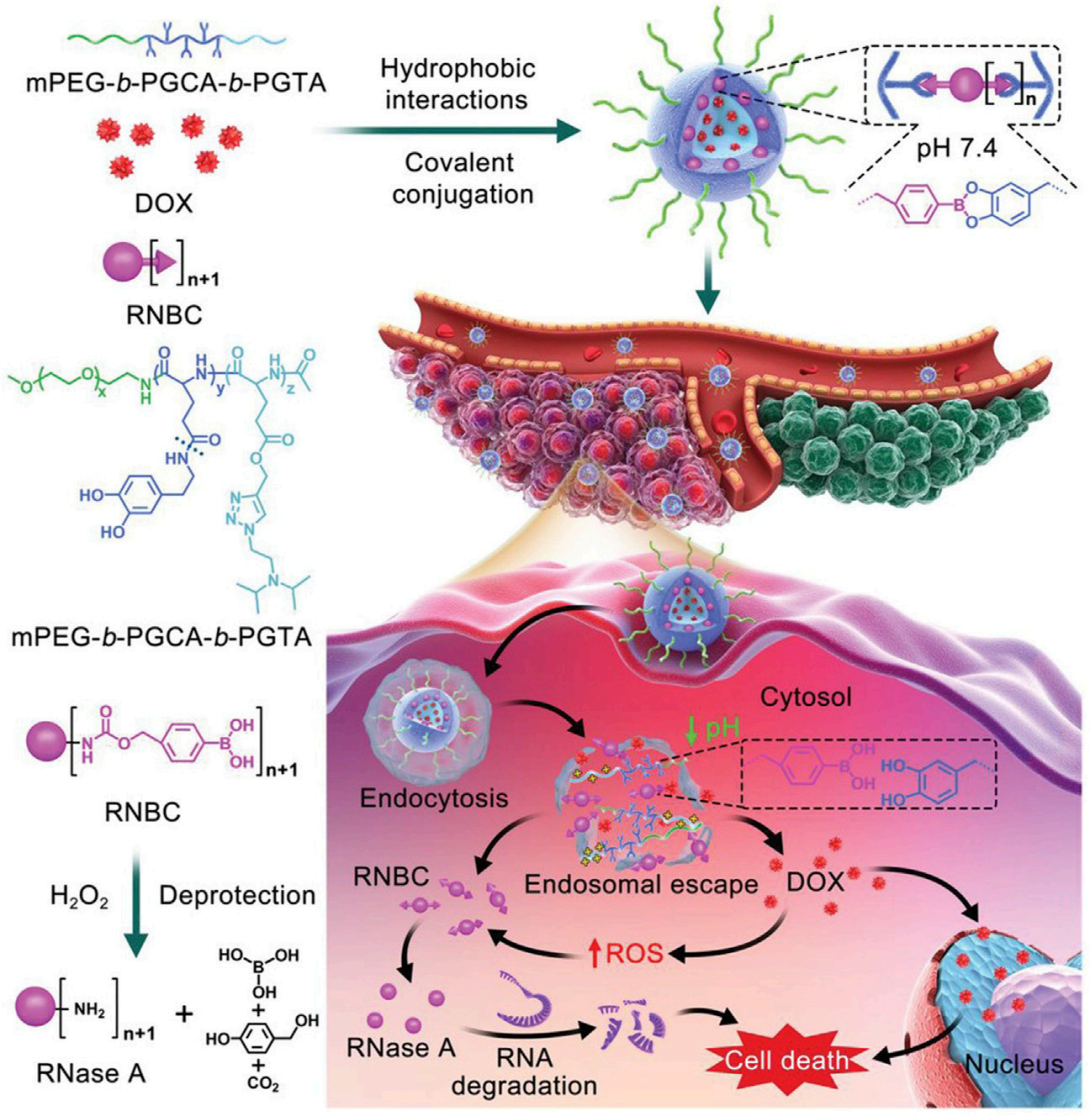
Schematic illustration of intracellular co-delivery of RNase A and Dox using a multistage cooperative drug delivery nanoplatform formed by mPEG-*b*-PGCA-*b*-PGTA for combination cancer therapy. Reproduced with permission from Ref. (Zhang et al., 2020).

In a recent study by Chen and others, a multistage cooperative nanoplatform was prepared by a well-designed triblock copolymer, mPEG-*b-*PGCA-*b*-PGTA, for the intracellular co-delivery of phenylboronic-acid-modified proteins and hydrophobic drugs (Figure 4) (Zhang et al., 2020). The mPEG-*b-*PGCA-*b*-PGTA contains a mPEG block, which could provide a hydrophilic domain and a shield from immune recognition, a middle PGCA block, which could be used for pH-reversible covalent conjugation of phenylboronic-acid-modified proteins to form dynamic lock-in micelles because of its catechol groups, and the terminal PGTA block that was used for pH-responsive release of hydrophobic drugs and endolysosomal buffering to mediate escape of proteins into cytosol. Specifically, hydrophobic Dox was loaded into the cores of micelles by hydrophobic interactions, and RNase A formed RNBC (RNase A modified with nitrophenyl tetramethyl-dioxaborolanyl benzyl carbamate groups), which was attached by reversible covalent conjugation.^43^ Based on circular dichroism, the structure of RNase A before and after covalent modification was consistent with that of natural RNase A, meanwhile, agarose gel retardation analysis and enzymatic reaction rate analysis proved that the activity of RNase A was not affected. In the synergistic anticancer experiment of tumor-bearing mice *in vivo* and TUNEL assay, NP-DOX-RNBC RNase A modified with nitrophenyl tetramethyl-dioxaborolanyl benzyl carbamate groups showed a stronger inhibitory effect on tumor growth than Dox and other groups, although NP-DOX-RNBC and Dox had similar cytotoxicity to B16F10 cells *in vitro*. The stability of the structure was verified *in vitro* and *in vivo*, indicating that this nanoplatform can effectively deliver proteins and small-molecule drugs into the cells.

A nanocarrier for co-delivery of photosensitizer chlorine e6 (Ce6) and CRISPR-Cas9 ribonucleoprotein (RNP) has also been reported to achieve the synergism of drug action. Nitrilotriacetic acid–disulfanediyldipropionate–polyethyleneglycol–*b*-polycaprolactone (NTA-SS-PEG-PCL) assembles into micellar nanoparticles (NTANPs), and hydrophobic photosensitizer Ce6 was encapsulated by its micellar core PCL block, HIS-tagged Cas9 RNP can be combined with the NTA-terminated PEG block on the micellar surface, then anionic micelles were coated with cationic iRGD modified copolymer. According to the experimental results *in vivo*, it is confirmed that there is a synergistic effect between Ce6 photodynamic therapy and Nrf2 gene editing (Deng et al., 2020b). This nanocarrier overcomes the problem of the uncontrolled release of Cas9 RNP from endocytosis nanoparticles, which provides a potential strategy for the co-delivery of gene-editing proteins and small-molecule drugs (Wang et al., 2017).

#### 2.3.2 Polymeric nanogels

Nanogels have been proved to have the advantages of small size, easy intracellular penetration, high stability in blood circulation, and good biocompatibility in various medical applications (Eckmann et al., 2014). Nanogels can encapsulate various bioactive compounds, such as hydrophilic and lipophilic drugs, DNA sequences, siRNA, peptides, and proteins. Depending on the structure of the nanogels and the interactions between drugs, drugs can be released by breaking the chemical bond or degrading the nanogels matrix (Hajebi et al., 2019).

**FIGURE 5:**
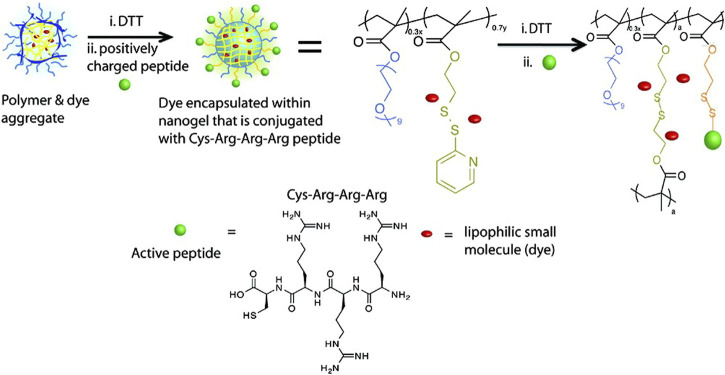
Structures of the nanogel’s polymer precursor and tri-arginine peptide. Reproduced with permission from Ref. (González-Toro et al., 2012).

A general strategy for nanocarriers combined with water-soluble proteins and lipophilic small molecules has been proposed by S. Thayumanavan and his colleagues, which is a self-assembled polymeric nanogel. β-galactosidase (β-gal, pI: 4.8) was used as the model protein, and 1,1′-dioctadecyl-3,3,3′,3′-tetramethyl-indocarbocyanine perchlorate (DiI) dye was used as the model small molecule (Figure 5). By using dithiothreitol (DTT) to initiate a thiol-disulfide exchange reaction between pyridyldisulfide (PDS) units, the self-assembly structure and lipophilic guest molecules were combined. Given that the surface of the nanogel contains charge-neutral functional groups, tri-arginine, which can provide a positive charge and show the characteristics of a cell-penetrating peptide, was used as a functional group to react with residual PDS to make it positively charged. Finally, it is bound to negatively charged protein by electrostatic interactions. Related studies have shown that nanogel can be effectively functionalized with cell-penetrating peptides, and the complex shows effective cellular uptake, in which lipophilic small molecules and proteins are internalized by cells at the same time. However, because proteins are bound to their surfaces by electrostatic interaction, the charge density on the surface of the nanogel will affect the binding efficiency of complementary charged proteins (González-Toro et al., 2012).

#### 2.3.3 Polymersomes

Polymersomes, also known as polymer vesicles, were made by self-assembly of amphiphilic block copolymers, predominantly with soft rubber hydrophobic segments (Chidanguro et al., 2018). The polymersomes have a core-shell structure, which wraps the hydrophilic molecules in the aqueous compartment and the hydrophobic molecules in the double layers of the vesicles (Mohammadi et al., 2017). Polymersomes can be designed with adjustable shape, fluidity, entanglement, permeability, stability, and responsiveness, and polymers can be customized according to appropriate physical properties or biocompatibility (Rideau et al., 2018). Polymersomes are increasingly being studied as carriers of imaging probes and therapeutic drugs because they can load large and small molecules that we are interested in (Leong et al., 2018).

A biodegradable chimeric polymersome was proposed by Zhong and his collaborators, based on asymmetric poly (ethylene glycol)-*b*-poly (ε-caprolactone)-*b*-poly (2-(diethylamino) ethyl methacrylate) (PEG-PCL-PDEA) triblock copolymers, which could load and transport protein and doxorubicin to the cytoplasm and/or cell nucleus at the same time (Liu et al., 2010). The PEG block was located on the surface of the polymersomes, providing good biocompatibility and stability for circulation, and the cationic PDEA blocks would be preferentially located inside the polymer, which was beneficial to the effective encapsulation and stability of proteins, and the hydrophobic PCL block was used to load DOX. During the formation of the polymer with pH 5.3, there was an effective electrostatic and/or hydrogen bond interaction between the exogenous proteins and the PDEA blocks, which may be the reason for the high protein loading of the chimeric polymer. The encapsulation of the protein did not significantly change the size distribution and zeta potential of the polymersomes, and the activity of the protein was preserved. *In vitro* studies suggested that the polymersomes could concurrently deliver proteins and Dox into the cytosol and cell nuclei.

#### 2.3.4 Polymeric nanocomplexes

Zhao and coworkers have developed a cationic polymeric nanocomplex to deliver cytotoxic proteins and drugs for use in combination cancer therapy. RNase A was used as the model protein. Firstly, the modification of phenylboronic acid to lysine residues blocked the activity of RNase A, forming phenylboronic-coupled compound RNBC. Then, in order to restore the biological function of the protein, glucose oxidase (GOD) was co-transferred to the nanocarrier to continuously produce hydrogen peroxide and restore the activity of RNase A. The final nanocomposite (PEI-oxliPt (IV)@RNBC/GOD) was formed by electrostatic interaction between cross-linked polyethyleneimine (PEI)-oxliPt (IV), RNBC and GOD (Lim et al., 2019). After accumulation in cancer cells, the nanocomplex can be restored to activated RNase A and cytotoxic platinum (II) drugs. RNase A inhibits cellular protein synthesis and active oxaliplatin inhibits DNA replication, leading to synergistic treatment of cancer (Hu et al., 2018).

**FIGURE 6:**
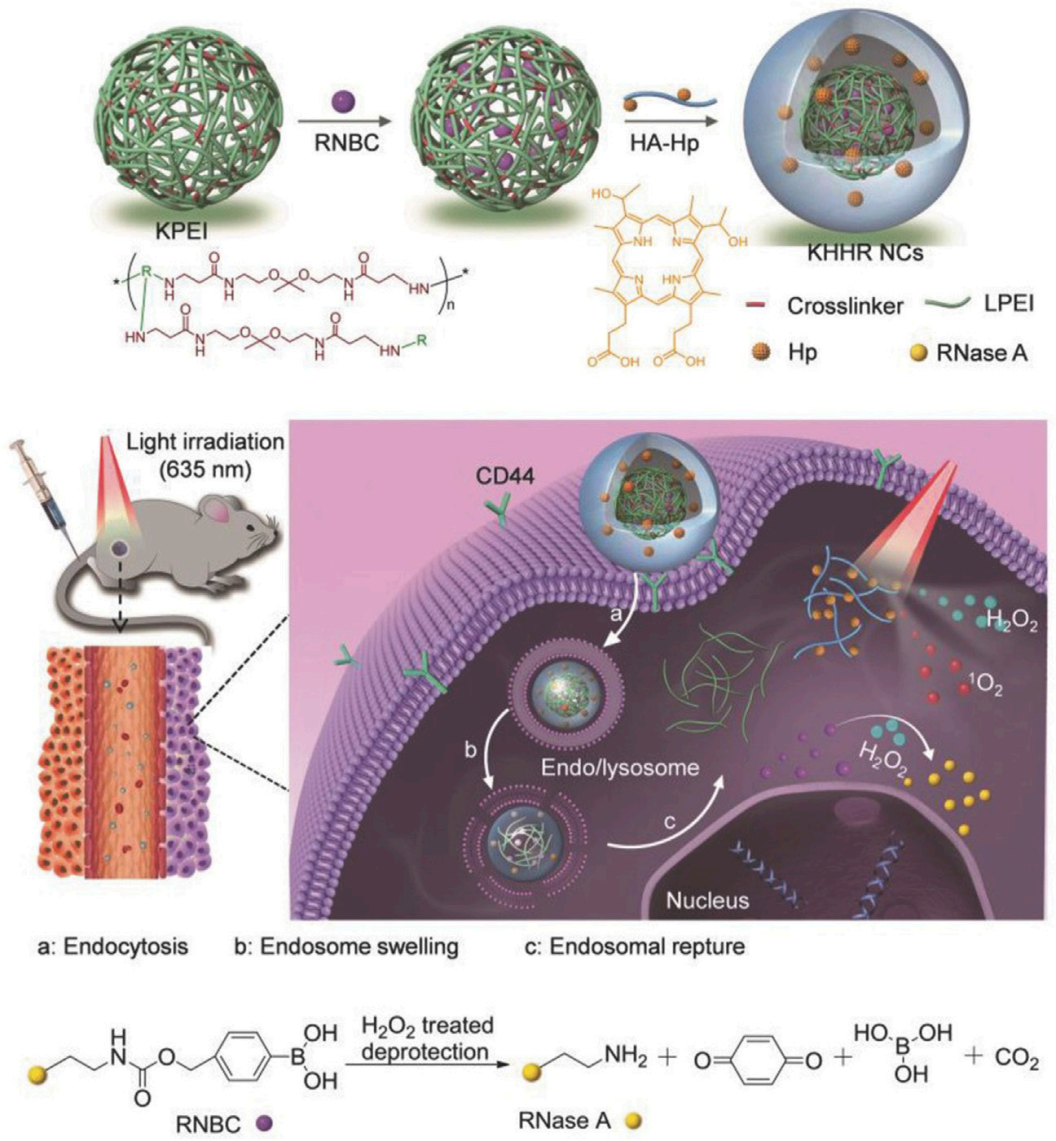
Schematic illustration of light-controlled protein delivery assisted by the “all-functions-in-one” nanocomplexes (NCs) toward synergistic cancer therapy. Reproduced with permission from Ref. (He et al., 2018).

Photodynamic therapy (PDT) has gradually become a new type of tumor therapy because of its low invasiveness, high cure rate, and little damage to the body, which has attracted great attention in preclinical research and clinical practice (Lucky et al., 2015; Zhang Y et al., 2018; Miao et al., 2019; Zhong et al., 2021; Zhang et al., 2022). In recent years, more and more tumor therapy systems combined with protein therapy and photodynamic therapy have emerged and achieved promising results. A multi-functional nanocarrier doped with photosensitizer (PS) was developed, and combined with the reversible protein engineering of reactive oxygen species (ROS) response, the targeted intracellular co-delivery of photosensitizer and protein was realized. In order to prepare the nanoparticles, the protein drug RNase A is immobilized on its lysine residue by H_2_O_2_ cleavable phenylboronic acid to form an inactivated prodrug RNBC (Figure 6). Cationic, acid degradable, ketal-crosslinked polyethyleneimine (KPEI) that form nanocomposite (NCS) with RNBC through electrostatic interactions, can improve the stability of protein and promote its internalization. The coupling of hyaluronic acid (HA) with hematoporphyrin (HP), namely PS (HA-HP), is further coated on the surface of NCS to enhance their serum stability by shielding positive charges, so that they can circulate in the blood for a long time, and promote tumor accumulation by the permeability and retention (EPR) effect. *In vitro* and *in vivo* results showed that the synergistic combination of PS-mediated photodynamic therapy and protein-mediated therapy achieved a strong anticancer effect compared with the control group (He et al., 2018).

### 2.4 Silica nanocarriers

Biomineralization-based nanoparticle drug delivery system has attracted wide attention in tumor therapy because of its simple preparation, good biocompatibility, degradability, easy modification, versatility, good targeting, and so on. As the main material for the biological application of silica, mesoporous silica nanoparticles have obvious advantages over other inorganic nanoparticles, such as adjustable porous structure, large specific surface area, easy surface functionalization and so on (Wan et al., 2013; Díez et al., 2016; Wang W et al., 2020). These unique properties enable mesoporous silica nanoparticles to load not only small-molecule drugs (Chang et al., 2010), but also proteins (Liu and Xu 2019), and siRNAs (Hom et al., 2010).In addition, the hydrophilicity of the surface makes it easier for silicon nanomaterials to be internalized by cells, and special porous forms can provide a protective environment for unstable molecules (Castillo et al., 2020).

A yolk shell nano-platform for co-delivery of tumor-specific cytochrome c (Cyt c) prodrug and doxorubicin was developed by Cai and his colleagues. The yolk-shell mesoporous silica nanoparticles (YMSNs) were used to load the water-soluble anticancer drug Dox, cytochrome c was immobilized on the surface of YMSNs by ROS cleavable borate ester bond. The results of the ABTS assay showed that the biological activity of Cyt c did not change through the binding and release process. The synergistic cytotoxic effects toward HepG2 cells through the co-delivery of Cyt c and Dox were proved by the cytotoxicity tests and apoptosis assay. The release of Cyt c can initiate the apoptosis pathway of mitochondria and Dox molecules can inhibit the nuclear DNA fragmentation induced by topoisomerase, which is considered to be involved in the killing process of tumor cells (Pei et al., 2018).

**FIGURE 7:**
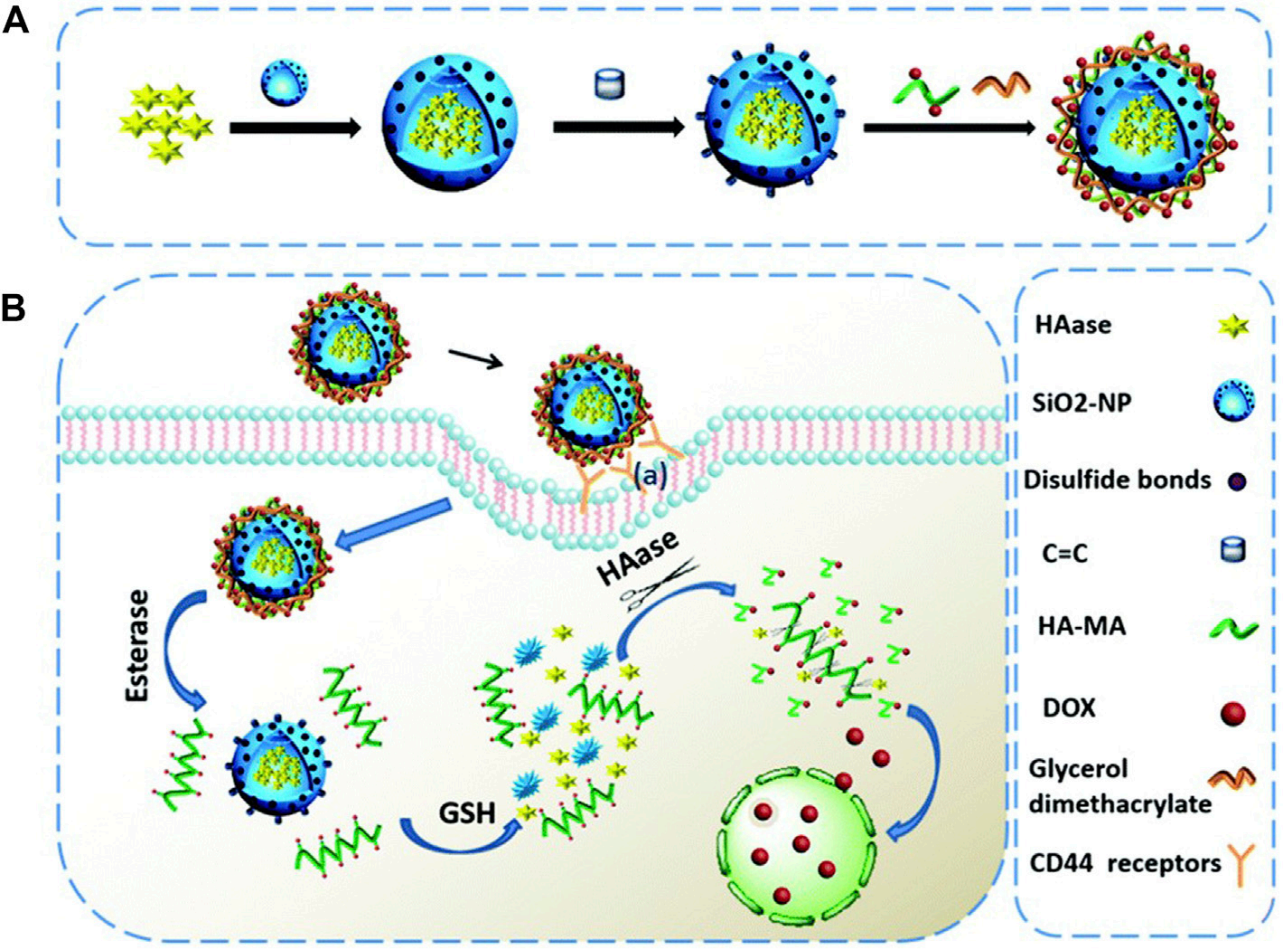
Schematic representation of **(A)** the fabrication of the HAase@SiO_2_@prodrug nanoplatform and **(B)** the tumor targeted cargo delivery, dual responsive cargo release and intracellular generation of cytotoxic antitumor drugs based on this nanoplatform. Reproduced with permission from Ref. (Chen et al., 2018).

The strategy of loading Dox into nanocarriers through chemical modification has always been an effective method, including conjugated modification, coupling with antibodies and so on (Shi et al., 2009; Zhu et al., 2011). For example, a multi-responsive nano-platform, containing hyaluronidase as the core, biodegradable silicon dioxide with disulfide bond as shell, and polyester-hyaluronic acid-adriamycin (PE-HA_1000k_-DOX) prodrug as crown, was synthesized. After CD44 mediated endocytosis, esterase and glutathione in tumor cells degraded polyester and silicon dioxide, respectively, releasing HA-DOX and hyaluronidase. Hyaluronidase catalyzed the decomposition of HA-DOX to produce highly toxic Dox to induce apoptosis of tumor cells (Figure 7). In this case, Dox is loaded into the prodrug by amidation, while hyaluronidase is loaded into the blurred core by physical encapsulation (Chen et al., 2018). This study suggests that co-delivery of enzymes and prodrugs based on the tumor response nano-platform may be a promising strategy for accurate tumor therapy.

In addition, nucleic acid targeted drug therapy is an efficient tumor therapy strategy, which has attracted wide attention (Okholm and Kjems 2016; Crooke et al., 2018). Recently, a nano-delivery system for dual targeting tumor therapy with intracellular RNA and nuclear DNA was developed by Ma and his colleagues, to achieve intracellular co-delivery of cis-platinum pro-drugs (DSP) and cytotoxic protein RNase A. They used large-pore mesoporous silica-coated β-NaYF_4_:20%Yb, 2%Er@β-NaGdF_4_(UCS) nanoparticles as carriers, which were prepared according to their previous work (Ding et al., 2018). First, UCS nanoparticles were modified with 3-Aminopropyltriethoxysilane. Then DSP was covalently conjugated with it to form UCSPt, and RNase A with macromolecular structure was loaded by physical adsorption to form the final product UCSPtR. The results of *in vitro* and *in vivo* studies showed that the prepared nanoplatform successfully delivered cytotoxic protein RNase A and DSP into tumor cells, and induced intracellular RNA degradation mediated and nuclear DNA targeted killing of tumor cells, showing a good therapeutic effect on the tumor (Teng et al., 2021).

Compared with normal cells, tumor cells accumulate a large amount of hydrogen peroxide (Lin et al., 2018). Therefore, carriers that co-deliver CAT and photosensitizers into cells to enhance their anti-tumor effect have been developed. For example, a multi-stage responsive intelligent nanoparticle system is designed to improve the efficacy of photodynamic therapy through intracellular delivery of CAT and photosensitizers (Yang et al., 2018). The water-soluble CAT were directly encapsulated in hollow silica nanoparticles by a simple reaction, and the photosensitizer Ce6, which was covalently conjugated to (3-aminopropyl) triethoxysilane, was doped into the silica lattice structure. Then the nanoparticles were modified with mitochondrial targeting molecule (3-carboxypropyl) triphenylphosphine bromide (CTPP), and further modified with acidic pH-responsive charge convertible polymers synthesized according to their previous work (Wang et al., 2013) by electrostatic interaction. The designed nanoparticles can not only encapsulate CAT and transport it into the tumor but also decompose tumor endogenous H_2_O_2_ to generate oxygen, overcome tumor hypoxia and greatly improve the therapeutic effect of PDT on solid tumors.

**FIGURE 8:**
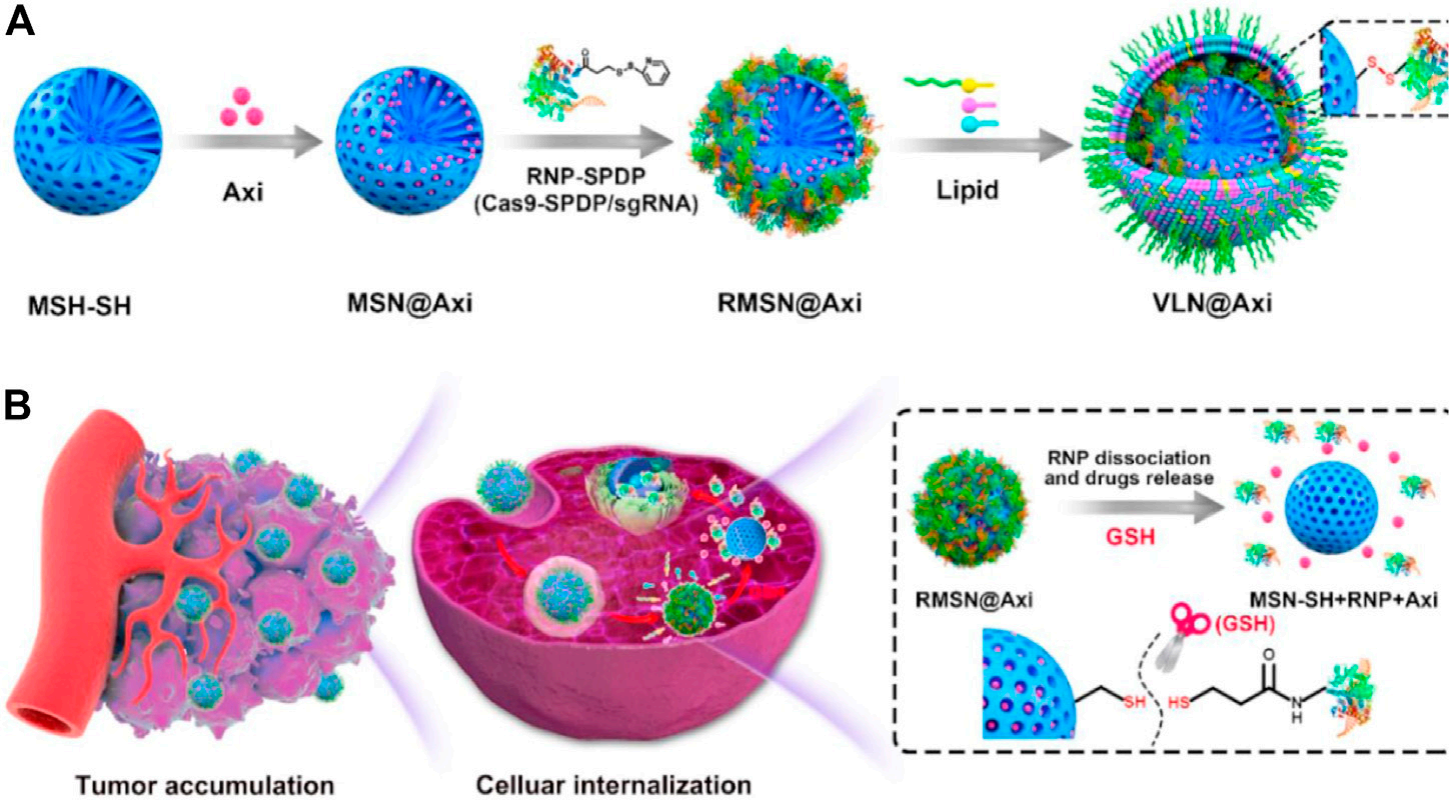
Schematic illustration for the synthesis of VLN@Axi **(A)** and delivery process **(B)** after intravenous injection. Reproduced with permission from Ref (Liu et al., 2020).

Interestingly, a virus-like nanoparticle (VLN), has been reported as a multi-functional nanoplatform, which can deliver CRISPR/Cas9 system and small-molecule drugs for effective malignant tumor therapy (Liu et al., 2020). The model drug, tyrosine kinase inhibitor Axitinib (AXI), was first loaded into the pores of surface vulcanized mesoporous silica nanoparticles (denoted as MSN-SH), and then RNP was connected to MSN-SH (denoted as RMSN) by a disulfide bond to lock the pores. Finally, liposomes containing PEG_2000_-DSPE were introduced to encapsulate RMSN to form VLN, to prolong the circulation and prevent RNP from being degraded by enzymes in the physiological environment (Figure 8). Results indicates that the VLN has great potential as a general platform for the development of advanced combined therapy for malignant tumors.

### 2.5 Metal-organic frameworks

The metal-organic frame (MOF) is a kind of hybrid porous material developed in recent years, which is composed of metal ions or clusters bridged by organic connectors (Wu and Yang 2017). MOF not only has various functions, high drug loading, and good biocompatibility but also has excellent adaptability of frame structure design and almost unparalleled surface adjustment, which makes it an ideal drug carrier (Huskić et al., 2016; Lian et al., 2017).

**FIGURE 9:**
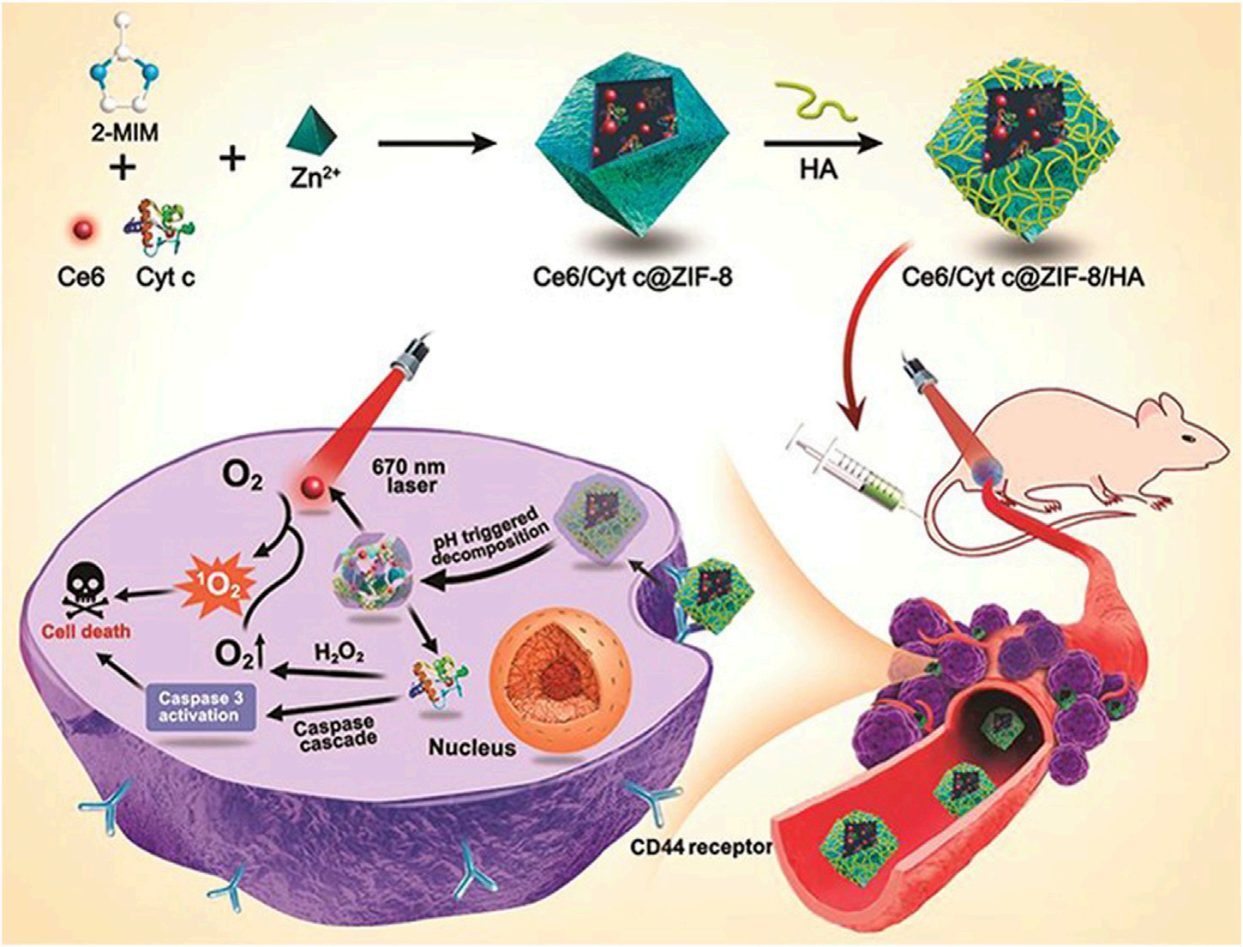
Illustration for the Synthesis of Ce6/Cyt c@ZIF-8/HA NPs and Their Applications in *Cancer* Cells Targeted Photodynamic and Protein Synergy Therapy. Reproduced with permission from Ref. (Ding et al., 2020).

Recently, a nano-platform based on MOF has been developed to achieve tumor cell targeted co-delivery of photosensitizers and therapeutic proteins to abtain collaborative photodynamic and protein therapy effects (Ding et al., 2020). Through a mild one-pot biomimetic mineralization process, the photosensitizer Ce6 and Cyt c were co-encapsulated in ZIF-8 nanoparticles to form a structure (Ce6/Cytc@ZIF-8) (Figure 9). Zn^2+^ ions interact with abundant carboxyl or amide bonds in proteins, and Ce6 is captured *in situ* in the pores of ZIF-8, which promotes the co-encapsulation of Cyt c and Ce6 into ZIF-8. Due to the small pore size of ZIF-8, the embedded protein and photosensitizer can not only reduce the leakage in the process of transportation but also protect the protein from being degraded by enzymes and ensure its activity. Using the coordination and electrostatic interaction between the carboxyl group and Zn^2+^ ions, the tumor-targeting ligand hyaluronic acid (HA) was coated on Ce6/Cytc@ZIF-8, and the nanoplatform (Ce6/Cyt c@ZIF-8/HA) was obtained. The experimental results *in vivo* and *in vitro* show that Ce6/Cytc@ZIF-8/HA nanoparticles have a good synergistic anticancer effect and low systemic toxicity, indicating that the MOF-based nano-platform is a promising strategy for intracellular co-delivery of proteins and drugs.

### 2.6 Other nanocarriers

With the development of nanotechnology, more and more kinds of drug delivery carriers are reported (Farokhzad and Langer 2009). In addition to the nanocarriers described above, some other nanocarriers that can realize the intracellular co-delivery of proteins and small-molecule drugs are also developed.

For example, a pH/H_2_O_2_ dual-responsive near-infrared fluorescence (NIRF) turn-on protein delivery system has been developed, which combines the NIRF turn-on probe and protein into a single magnetic nanoparticle (MNP)-based nanocarrier. QCy7 and trypsin were used as model agents for the study. Active fluorophore QCy7 can be coupled with phenylboronic acid (PBA) to form QCy7-PBA and can be released in the presence of hydrogen peroxide. Trypsin was chemically modified with PBA to form Try-PBA. Using polydopamine (PDA) coated MNP (MNP@PDA) as a scaffold, MNP@PDA@PEG/SHA was obtained by mixing with SHA (salicyl hydroxamates)-NH_2_ and PEG-SH, respectively. QCy7-PBA and Try-PBA are covalently linked to MNP@PDA@PEG/SHA through complexation between SHA and PBA, thus MNP@PDA@PEG/SHA-Q/T is successfully prepared. Under the intracellular condition of low pH and high level of H_2_O_2_, the system can realize the controlled release of active protein and mediate the restoration of QCy7 fluorescence to monitor the intracellular protein delivery (Xu et al., 2019).

**FIGURE 10:**
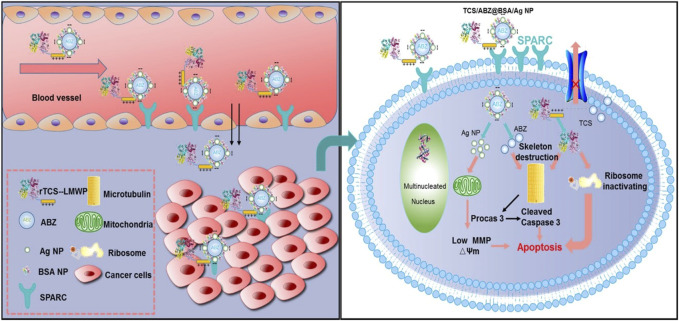
Schematic illustration of tumor delivery and synergistic effect *via* co-delivery of TCS and ABZ by silver nanoparticles. Reproduced with permission from Ref. (Tang et al., 2017).

As a natural polymer, albumin is highly attractive because of its non-toxicity, non-immunogenicity, biodegradability, and biocompatibility (Tan and Ho 2018). Albumin also has received special attention as a drug carrier because of its affinity to albumin receptors on tumor cells, such as SPARC and gp60 (Desale et al., 2018). A self-assembled bovine serum albumin (BSA)-based nano-system has been developed for the combined delivery of trichosanthin (TCS) and albendazole (ABZ) to overcome multidrug resistance and metastasis (Figure 10). TCS is a type I ribosome-inactivating protein, which has high anti-tumor activity through the mechanism of ribosome inactivation and apoptosis, while ABZ is a β-tubulin inhibitor, which shows strong anti-tumor activity. Firstly, ABZ is physically encapsulated in albumin-coated silver nanoparticles (called ABZ@BSA/Ag NP), and then a recombinant cell-penetrating TCS (referred to as RTL) was loaded by charge interactions between negatively charged ABZ@BSA/Ag NP and cationic RTL to form the nano-system RTL/ABZ@BSA/Ag NP. *In vitro* experiments suggested that this co-delivery system inhibited the proliferation of drug-resistant A549/T and HCT8/ADR cells. Furthermore, *in vivo* studies indicated that RTL/ABZ@BSA/Ag NP effectively inhibited the tumor metastasis to the lung (Tang et al., 2017).

Interestingly, Geest and coworkers synthesized transiently responsive BSA-polymer conjugates through a “grafting-from” reversible addition–fragmentation chain transfer (RAFT) polymerization of a dioxolane-containing acrylamide with a BSA-based chain transfer agent (CTA) (Vanparijs et al., 2015). The as-prepared BSA-polymer conjugates could self-assemble into nanoparticles at physiological pH and temperature. Hydrophobic small molecule immune-modulators could be loaded into the hydrophobic cores of the nanoparticles. Under acidic conditions, the hydrolysis of hydrophobic dioxolane groups into hydrophilic diol units resulted in the disassembly of nanoparticles and the release of small drugs. Therefore, this platform holds great application potential as intracellular co-delivery system of proteins and hydrophobic drugs.

In addition, an organic/inorganic nanocomposite system targeting mitochondria was developed to simultaneously load and deliver Ce6 and hydrophilic CAT for improved PDT activated by NIR laser. For the preparation of this system, oleic acid (OA) capped upconversion nanoparticles (UCNPs) were first prepared. Then using the double bond of oleic acid ligands, the active azide group was directly introduced by the thiol-ene click reaction, thus the hydrophobic pocket was retained on the periphery of the nanoparticles, and the hydrophobic photosensitizer Ce6 was loaded into the hydrophobic pocket composed of oleic acid molecules. Dendrimers can be further grafted onto UCNPs by azide-acetylene click reaction, thus constructing hydrophobic and hydrophilic “pockets” in a single nanoparticle at the same time. CAT was encapsulated in hydrophilic dendrimers with tertiary amine groups due to electrostatic interaction. The surface of dendrimer contains a large number of hydroxyl functional groups, which realizes the further surface modification of nanoparticles by mitochondrial targeting molecule (3-carboxypropyl) triphenylphosphonium bromide (CTPP). This co-delivery system significantly improved PDT efficacy by CAT catalysis-mediated alleviation of tumor hypoxia and CTPP-mediated mitochondria targeting upon irradiation using 980 nm laser (Liang et al., 2020).

**FIGURE 11:**
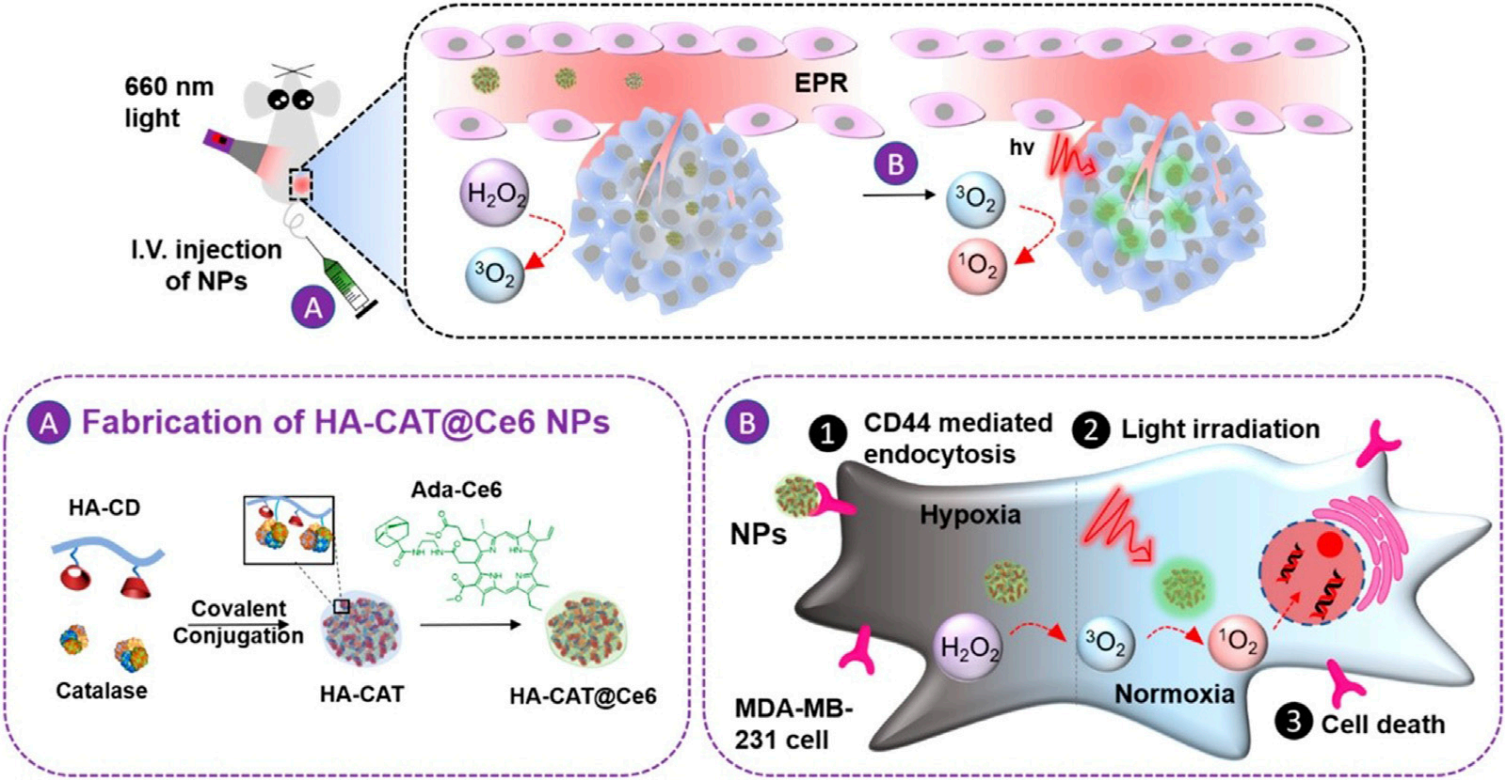
Schematic illustration of the preparation of HA-CAT@aCe6 NPs and the processes after intravenous injection into tumor-bearing mice. Reproduced with permission from Ref. (Phua et al., 2019).

Zhao et al. developed another strategy for intracellular co-delivery of CAT and Ce6. Firstly, CAT was covalently coupled to hyaluronic acid that was functionalized with β-cyclodextrin to form HA-CAT NPs. Then Ce6 was modified with adamantane to obtain adamantine-modified Ce6 (aCe6). The aCe6 could be encapsulated into HA-CAT by supramolecular ways between β-cyclodextrin and adamantane to obtain HA-CAT@aCe6 (Figure 11). *In vitro* and *in vivo* experiments showed that HA-CAT@aCe6 showed great potential in overcoming hypoxia of photodynamic therapy (Phua et al., 2019). The advantage of this supramolecular loading mode is that it is controllable and predictable, and it can adjust the loading ratio between photosensitizer and CAT because it enables different components to be stored separately (Webber and Langer 2017).

**FIGURE 12:**
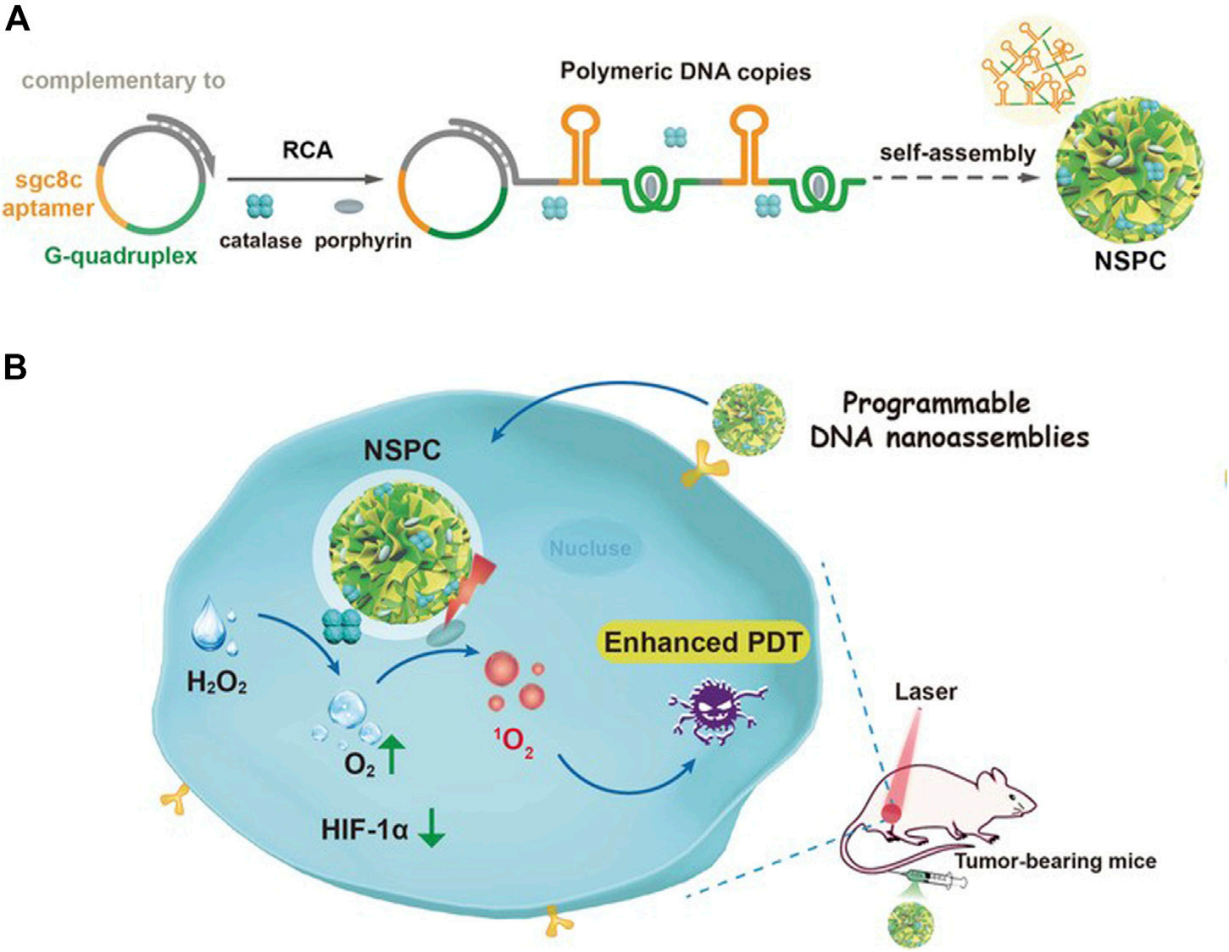
Illustration of designed DNA nanoassemblies for safe and effective PDT. **(A)** Design and synthesis of DNA nanosponges. **(B)** Proposed strategy for enhanced PDT with programmable nanoassemblies. Reproduced with permission from Ref. (Pan et al., 2020).

Moreover, Liu and coworkers prepared a programming DNA nanoassembly to simultaneously deliver CAT and porphyrin (Pan et al., 2020). DNA nanoassemblies with multiple functions were constructed by rolling circle amplification (RCA) with a circular DNA template complementary to sgc8c aptamer and G-quadruple. Sgc8c aptamer targets cancer cells overexpressing tyrosine-protein kinase 7 (PTK-7), and G-quadruplex can intercalate with photosensitizer porphyrin. In the process of RCA reaction, photosensitizer and CAT were added at the same time to prepare dual-drug-loaded DNA nanosponges NSPC (Figure 12). The assembled DNA nanoparticles can effectively load photosensitizers and CAT, and accurately target tumor cells. Upon being internalized by cancer cells, NSPC catalyzed endogenous hydrogen peroxide into oxygen, relieving the hypoxic tumor microenvironment, resulting in enhanced photodynamic therapy.

## 3 Conclusion and prospects

In this review, we summarize the recent intracellular co-delivery systems of proteins and small-molecule drugs and their applications in tumor therapy. Obviously, the combination of proteins and small-molecule drugs, such as therapeutic proteins/chemotherapeutics, therapeutic proteins/photosensitizers, and CAT/photosensitizers, results in enhanced synergistic anti-tumor effects in preclinical trials. However, the clinical applications of these systems remain elusive due to the existence of several challenges.

First, the heterogeneity of the materials and the complexity of various nanocarriers result in the difficulty in reproducible and large-scale preparation of optimal intracellular co-delivery systems of proteins and drugs for clinical translation (Zheng et al., 2021b). Therefore, new synthesis methodologies need to be developed to synthesize novel materials with high homogeneity and precise characterization. Meanwhile, simplification should be kept in mind during the design of new intracellular co-delivery systems in order to promote their market arrival by easing upscaling and reducing costs. In addition, due consideration needs to be given to the availability and cost of the desired proteins and drugs in the co-delivery systems during the clinical conversion process. It can not only achieve the treatment of a variety of diseases, but also achieve the integration of diagnosis and treatment.

Second, proteins and small-molecule drugs loaded on nanocarriers can be accurately delivered to the tumor site. Although it can be targeted by the specificity of the tumor site or tumor cells, it can also be driven by some external conditions, such as magnetic field, ultrasound, or light and heat, so that the nanocarriers can release drugs at the tumor site (Yang et al., 2018; Wang et al., 2019; Wang Xet al., 2020; Dai et al., 2021; Zheng et al., 2021c). However, it is difficult to guarantee the effective rate and activity release of small molecular drugs and proteins to the tumor site. Recently, it has been reported that nanocarriers are modified by cell membranes or exosomes, these functionalized and biologically recognized nanocarriers greatly improve the membrane permeability and bioavailability, which also provides an idea for the design of co-delivery carriers for proteins and small-molecule drugs (Fu et al., 2020; Zhu et al., 2022).

Third, proteins and small-molecule drugs need to be loaded and released in a controlled manner, which requires in-depth exploration of the physical and chemical properties of proteins and small drugs, and making full use of the different structural properties of both agents, including their specific functional groups, hydrophilicity/hydrophobicity, charge density and so on. The rational applications of chemical or physical methods are able to endow the nanocarriers a controlled drug release profile, such as reversible covalent modification, electrostatic interactions, hydrophobic interactions, hydrogen bond, and van der Waals force.

Fourth, the proteins and small-molecule drugs co-loaded in the intracellular co-delivery system need to be thoroughly biologically evaluated to ensure that they enter the cell to be active and to avoid potential adverse reactions, which requires a full understanding of the molecular and cellular mechanisms involved. The protein needs a complete structure to exert its activity, and the covalently modified protein is more stable in the process of delivery, but it is difficult to restore its activity in the cell. The strategy of restoring the activity of modified proteins through stimuli-responsive cleavage of covalent bonds in cells is a promising way. Meanwhile, the endosomal entrapment of protein therapeutics will induce the enzymatic degradation of proteins, which requires the development of more potent endosomal escape strategies.

With the development of chemistry, material technology and nanomedicine, the emerging of new design concepts, and the deeper insights into molecular and cellular mechanisms involved in intracellular drug delivery, we believe that new intracellular co-delivery systems that can address the above challenges will be developed to further improve the combined anticancer effects of various combinations of proteins and small molecular drugs and promote their clinical translation in the cancer treatment.
